# Research progress on blood compatibility of hemoperfusion adsorbent materials

**DOI:** 10.3389/fbioe.2024.1456694

**Published:** 2024-10-01

**Authors:** Liangqing Zhang, Guohao Liu, Qingping Xia, Li Deng

**Affiliations:** ^1^ Department of Anesthesiology, The Second Affiliated Hospital of Guangdong Medical University, Zhanjiang, Guangdong, China; ^2^ Department of Medical Imaging, Affiliated Hospital of Jilin Medical University, Jilin, China; ^3^ Department of Science and Education, Gaozhou People’s Hospital, Gaozhou, Guangdong, China; ^4^ Department of Cardiovascular Surgery, Gaozhou People’s Hospital, Gaozhou, Guangdong, China

**Keywords:** hemoperfusion, adsorbent materials, blood compatibility, research progress, biocompatibility

## Abstract

This comprehensive review examines the latest developments in improving the blood compatibility of hemoperfusion adsorbents. By leveraging advanced coating and modification techniques, including albumin-collodion, cellulose, hydrogel, and heparin coatings, notable enhancements in blood compatibility have been achieved across diverse adsorbent types, such as carbon-based, resin-based, and polysaccharide-based materials. Despite promising laboratory results, the intricate manufacturing processes and elevated costs present significant challenges for broad clinical application. Therefore, future endeavors should focus on cost-benefit analysis, large-scale production strategies, in-depth exploration of blood-material interactions, and innovative technologies to propel the development of safer and more effective blood purification therapies.

## Introduction

Hemoperfusion technology is an efficient blood purification strategy that eliminates harmful substances from the bloodstream using specialized extracorporeal adsorbent materials. This approach mimics the body’s natural detoxification processes and plays a crucial role in enhancing patient safety (Ricci et al., 2022; Ronco and Bellomo, 2022). With advancements in science and technology, the variety of adsorbent materials has significantly expanded, improving their performance and facilitating the development of hemoperfusion methods.

The selection of adsorbent materials is critical; ideal candidates must possess safety, non-toxicity, mechanical strength, stable chemical properties, low allergic reactions, and excellent blood compatibility. Significant differences in biocompatibility exist among materials like activated carbon, silica gel, resins, and carbon nanotubes (Li et al., 2019).

Blood compatibility is vital for the clinical efficacy of hemoperfusion adsorbents, as it minimizes adverse reactions with blood components and enhances the longevity of the materials (Wang et al., 2021; Gao et al., 2022; Damianaki et al., 2023).

The chemical properties and physical structure of adsorbents are foundational to achieving optimal biocompatibility (Nakane, 2004; Weber et al., 2018). Specifically, surface chemistry, hydrophilicity, and biodegradability directly influence interactions with blood components, thereby affecting overall biocompatibility (Maitz et al., 2019; Li et al., 2023). Recent studies highlight the importance of refining surface chemistry to improve both toxin adsorption and blood compatibility. Furthermore, physical attributes such as porosity and particle size significantly impact blood flow and interactions with blood cells (Wu et al., 2023; Bhattacharjee et al., 2024). Understanding the interactions between adsorbent materials and blood components remains a key research area. These interactions affect clinical safety by influencing platelets, coagulation factors, and immune cells (Zhang et al., 2023). Researchers can utilize surface modification techniques to reduce adverse effects on blood components and enhance biocompatibility, employing various experimental methodologies such as *in vitro* cultures and clinical trials (Zhou et al., 2023). As research progresses, novel adsorbents, such as protein-polysaccharide complexes and metal-organic frameworks (MOFs), are emerging. MOFs, with their high surface area and tunable pore structures, represent a promising frontier in hemoperfusion (Gan et al., 2021).

In recent years, review studies on hemoperfusion adsorbent materials have primarily focused on aspects such as material types, modification techniques, and biocompatibility evaluations. However, these reviews tend to emphasize the performance and applications of the materials, while lacking in-depth exploration of how modification techniques specifically influence the mechanisms of blood compatibility. This article aims to bridge this gap by systematically reviewing the latest research progress on various modification techniques (such as surface coating and chemical modification) for enhancing the blood compatibility of adsorbent materials. It will provide an in-depth analysis of their mechanisms of action and offer new insights and directions for future research.

### Understanding blood compatibility and coagulation mechanisms for hemoperfusion adsorbents

Blood compatibility is a critical factor for assessing biomedical materials, defined as a material’s ability to avoid adverse reactions upon contact with blood (Bowry et al., 2021). Materials with poor blood compatibility can trigger harmful physiological responses, such as thrombosis, hemolysis, and immune reactions, undermining the functionality of medical devices and posing significant health risks to patients (Sharma, 2001). Thus, developing materials with excellent blood compatibility is essential in biomedical engineering (Brash et al., 2019).

**FIGURE 1:**
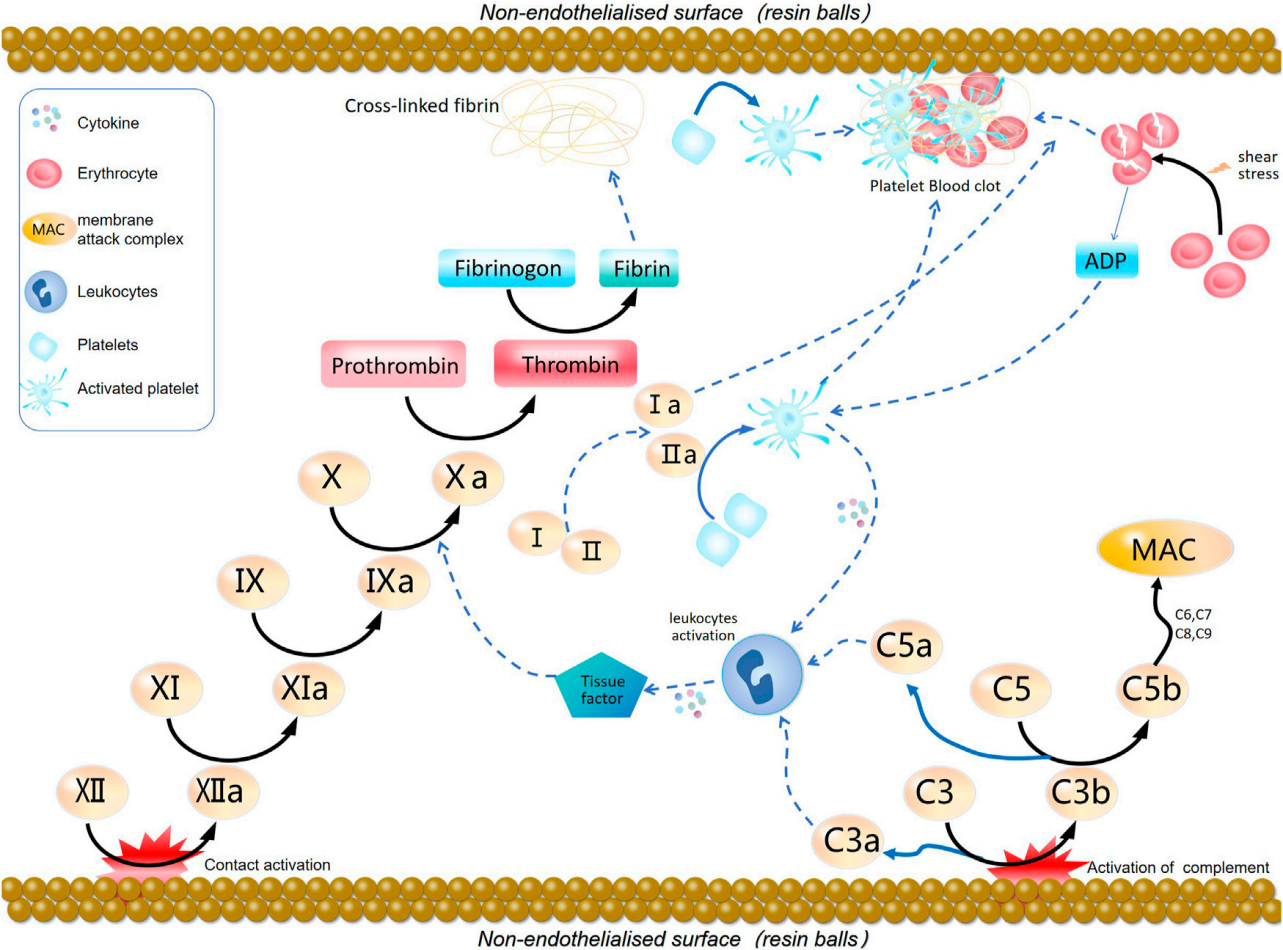
Blood adsorption material-induced coagulation cascade reaction mechanism diagram. The contact surface is responsible for generating activation factor XII, which initiates the intrinsic coagulation pathway, leading to the generation of thrombin and the conversion of fibrinogen into cross-linked fibrin. Thrombin also acts as a platelet agonist, causing platelet activation and aggregation. The complement pathway is activated on the surface of the biomaterial, enhancing the common coagulation pathway. The adhesion and activation of platelets on the foreign surface release platelet agonists, which, along with C3a and C5a from the complement pathway, directly activate receptors on the surface of inflammatory cells, triggering the release of cytokines. This stimulates the extrinsic coagulation pathway, enhancing the formation of thrombin and clot formation. Red blood cells undergo shear-induced fragmentation, releasing adenosine diphosphate. (ADP), which activates platelets.

The coagulation pathway, which leads to clot formation after injury or foreign material exposure, involves two main routes: the intrinsic and extrinsic pathways. The intrinsic pathway initiates from interactions between specific blood factors and foreign materials, involving factors XII, XI, IX, and VIII. Conversely, the extrinsic pathway begins with tissue factor (TF) binding to factor VII, engaging factors VIIa and Xa (Camire, 2021). Both pathways ultimately activate factor X, converting it to thrombin, which triggers fibrin clot formation (He et al., 2021). Materials with inadequate blood compatibility may activate either pathway, promoting harmful coagulation reactions and thrombus formation. Understanding and effectively modulating these activation mechanisms is essential for developing superior blood-compatible materials. The mechanism of coagulation cascade induced by blood adsorption materials is illustrated in Figure 1.

During hemoperfusion, adsorbent materials directly interface with blood, which can activate the coagulation system due to their non-physiological properties, potentially leading to thrombosis or hemolysis (Kuchinka et al., 2021). To ensure safety and efficacy, high-performance adsorbents must adhere to stringent requirements (Erturk et al., 1987; Chandy and Sharma, 1993):

1) Non-toxicity to Humans: The material must not provoke allergic reactions, immune responses, or any harmful effects upon contact with blood.
2) Excellent Biocompatibility: It should not induce hemolysis, coagulation, or other adverse reactions.
3) Stable Chemical Properties and Mechanical Strength: The material must maintain chemical stability, resist fragmentation, and avoid damaging blood cells throughout the hemoperfusion process.
4) Superior Adsorption Performance: The material should have a rapid adsorption rate and high capacity.

### Enhanced blood compatibility of hemoperfusion adsorbents through advanced coating technologies

Coating technology plays a crucial role in improving the blood compatibility of materials used in blood perfusion adsorption (Pagel and Beck-Sickinger, 2017). This technology utilizes methods such as physical adsorption, chemical bonding, and surface modification to create protective coatings. These coatings prevent the adhesion of blood proteins and cells, reducing coagulation and immune responses, thereby enhancing biocompatibility (Wei and Haag, 2015).

The selection of coating technology is guided by the substrate characteristics and intended application. Carbon-based materials, like graphene and carbon nanotubes, are favored for their high conductivity and chemical stability, often coated using chemical vapor deposition (CVD) and physical vapor deposition (PVD) (Rokosz et al., 2020). Resin-based materials, such as polyurethane and polyethylene, are commonly found in medical devices and are typically coated via spraying or dip coating (Chen et al., 2021). Polysaccharide-based materials, including chitosan and hyaluronic acid, are popular in drug delivery and tissue engineering, often utilizing microencapsulation and layer-by-layer assembly (Redolfi Riva et al., 2022).

Effective coating technology must ensure good blood compatibility and adsorption efficiency. Therefore, choosing the right coating involves evaluating both substrate properties and application requirements. Advancements in coating technologies have demonstrated the potential to enhance not just biocompatibility but also add properties like antibacterial and anti-inflammatory effects (Wen et al., 2024).

Key considerations in implementing coating strategies include (Ursino et al., 2022; Xiong et al., 2024):

Material Selection: The choice of coating materials is vital; for instance, electroporation technology can apply coatings that enhance anti-inflammatory and antibacterial properties.

Process Control: Conditions such as temperature, pH, and reaction time must be controlled to ensure uniform coatings and stability. High-molecular-weight polymers can promote even particle deposition, avoiding issues like the coffee-ring effect.

Performance Metrics: Biocompatibility and anti-biofouling capabilities are critical measures of coating effectiveness. Antifouling peptide coatings, for example, have shown substantial stability and efficacy in biological fluids.

Microencapsulation technology is effective for sustained, targeted drug delivery, particularly for porous polysaccharide-based materials (Bale et al., 2016). While it has advantages like preventing leakage and controlling adsorption rates, its complexity and cost pose challenges (Ma, 2014).

Surface modification alters substrate properties to improve biocompatibility and functionality, mainly suited for smooth resin-based materials (Ren et al., 2015). However, its effectiveness may be limited by the material’s chemical nature (Sun et al., 2020).

Composite preparation combines different materials to enhance mechanical properties and durability, though the complexity of preparation and compatibility issues must be considered (Xing et al., 2023).

Research by Qiang Wei et al. emphasizes the significance of coating-substrate interactions, crosslinking, and functional design in improving stability (Wei and Haag, 2015). Innovations in universal coatings and bioinspired technologies have expanded applications and enhanced blood compatibility and adsorption efficiency.

Large specific surface area substrates, such as porous spheres and nanofibers, are prevalent in adsorption materials due to their unique structural properties (Dong et al., 2024). Porous spheres possess a high specific surface area and favorable pore structure, enhancing adsorption efficiency and capacity (Zhao et al., 2023). Nanofibers, prepared via electrospinning technology, exhibit high specific surface areas and excellent mechanical properties, making them versatile for filtration and adsorption applications. The incorporation of these large specific surface area substrates into adsorption materials can significantly improve their performance.

Applying coating technology to large specific surface area substrates can further enhance their adsorption performance and stability. For example, coating the surface of porous spheres with functional polymers can improve their adsorption capacity for organic pollutants (Zhao et al., 2023). Simultaneously, coating nanofibers with antibacterial layers can significantly enhance their antibacterial performance and biocompatibility (Jo et al., 2014). Such application examples illustrate that careful selection and optimization of coating technologies can substantially improve the adsorption performance and efficacy of large specific surface area substrates.

Heparin-mimetic coatings - a form of biomimetic coating technology - enhance the blood compatibility and anticoagulation properties of adsorption materials by applying substances akin to heparin onto their surfaces (Riesenfeld et al., 1995; Biran and Pond, 2017). This technology has wide applications in blood purification and medical devices, significantly minimizing thrombosis and inflammatory responses while enhancing material safety and biocompatibility. The use of heparin-mimetic coatings introduces novel strategies for utilizing adsorption materials in the biomedical field (Patel, 2021).

Novel hydrogel microsphere blood adsorbents are characterized by their high specific surface area and excellent biocompatibility (Song et al., 2021). Coating the surfaces of these hydrogel microspheres with specialized functional coatings can greatly enhance both their adsorption performance and stability (Wu et al., 2020). With favorable mechanical properties and biocompatibility, these materials find extensive application in blood purification and medical devices. Recent studies indicate that the application of functional coatings on hydrogel microspheres can significantly augment their adsorption capacity for toxins and pathogens (Buhrman et al., 2013).

While numerous adsorption materials are available, activated charcoal, resins, and polysaccharides are prevalent in blood perfusion applications. An in-depth understanding of their biocompatibility is essential for optimal coating material selection, as discussed in the following sections.

### Characteristics and improvement of blood compatibility of inorganic adsorbents

#### Carbon-based materials

Carbon-based materials, obtained through the carbonization of organic precursor materials, are porous and have received considerable attention in the field of blood perfusion due to their unique properties. These materials primarily include activated carbon, mesoporous/multi-level porous carbon-based materials, carbon nanotubes, and graphene-based materials. Known for their high specific surface area and excellent adsorption performance, they effectively remove a variety of toxin molecules (Gupta et al., 2019). Activated carbon, the earliest carbon-based material used in blood perfusion, is currently widely used in clinical practice (Lie et al., 1976; Cheah et al., 2017). However, it exhibits poor blood compatibility and mechanical strength, which can lead to adverse reactions such as hemolysis, coagulation, and microthrombosis. To enhance its blood compatibility, surface coating technology is often employed. The choice of embedding materials is crucial as it affects the adsorption rate, necessitating the selection of appropriate coating materials (Davenport, 2017). Given the diversity of activated carbon coating technologies, this article discusses several representative ones.

#### Albumin-collodion coating technology in carbon-based materials

Espinosa-Meléndez utilized the principle of artificial cells to create an ultra-thin film of albumin-collodion coated activated carbon directly on the surface of individual particles, thereby microencapsulating the carbon. This method seals the particles with a membrane that prevents any free powder from entering the bloodstream while retaining platelets. Activated carbon coated with a collodion (nitrocellulose) membrane exhibits sufficient blood compatibility, making it suitable for treating uremia and acute poisoning. *In vivo* experiments have shown that, even in cases of platelet sensitivity (such as liver failure), adsorbing albumin onto collodion achieves high blood compatibility with a low platelet destruction rate. Therefore, the research team believes that albumin-collodion coated activated carbon is an ideal coating membrane material (Chang, 1984). This coating technology has demonstrated promising results in enhancing the blood compatibility of activated carbon.

#### Cellulose nitrate and cellulose acetate coating technologies in carbon-based materials

Cellulose nitrate and cellulose acetate are commonly used coating materials. To determine which coating is more effective, the Denti E team conducted a series of experiments using Norit RBXS 1 activated carbon with different coating treatments (Denti et al., 1975). The trials were divided into three groups: cellulose acetate, cellulose acetate + formamide, and cellulose acetate + KOH. Despite having the same thickness, these cellulose acetate coatings had varying structures due to different treatment methods.

To evaluate the performance of these coating materials, the researchers used a closed recirculation system. They tested coated and uncoated charcoal particles (20 g) in this system using an aqueous solution containing creatinine (initial concentration of 100 mg/L), uric acid (initial concentration of 500 mg/L), and vitamin B-12 (initial concentration of 20 mg/L). The charcoal particles were placed in a cylindrical column. Simultaneously, to assess the impact on blood, they used bovine blood in the same artificial circuit to test platelet loss. The experiment was conducted at a constant system temperature of 37°C with a flow rate of 200 mL/min.

The *in vitro* experimental results showed that the hematocrit remained constant during the experiment, except for the uncoated carbon. No platelet loss was observed in the empty column. However, in the uncoated charcoal-filled column, platelet loss was as high as 70%–80%. In contrast, cellulose acetate treated with KOH caused the greatest platelet loss, which was consistent with previous discussions on membrane structure. Considering the simplicity of the coating process and the high toxin removal rate, cellulose nitrate coating appears to be the preferred choice (Denti et al., 1975; Pişkin et al., 1981).

#### Hydrogel as a coating material in carbon-based materials

Research progress on hydrogels as a coating technology for activated carbon has not been smooth. Back in 1971, Andrade et al. suggested that hydrogels, as a coating material applied to activated carbon, could enhance its mechanical strength, improve adsorption performance, facilitate processing and handling, provide waterproof protection, adapt to various substrates, and improve regeneration performance. These advantages make hydrogel coatings have broad prospects and potential in the application of activated carbon (Andrade et al., 1971). However, research and development progress seemed slow until 2017 when Cai et al. (2017). developed activated carbon with a zwitterionic polycarboxybetaine (PCB) hydrogel coating, marking a breakthrough in this technology. Zwitterionic hydrogels are water-rich polymer networks composed of zwitterionic materials, which exhibit excellent anti-biofouling properties, bio/blood compatibility, high water content, and large mesh sizes. They provide mechanical strength similar to biological tissues and high permeability to molecules, making them an ideal coating material for activated carbon. This research also confirmed that the PCB hydrogel coating significantly improved the blood compatibility of activated carbon, greatly stimulating interest in the research and development of hydrogels. In 2018, Zhang L used polycarboxybetaine methacrylate hydrogel (pCBMA) to modify activated carbon (AC) to improve its biocompatibility and adsorption capacity in biological environments. A self-made device was used to prepare hydrogel beads and hydrogel-coated activated carbon (pCBMA-AC) with controllable sizes, and the preparation conditions were optimized. The physical and biological properties of pCBMA-AC with different diameters were then studied. The research confirmed that 2 mm pCBMA-AC exhibited good stability, with a leakage rate of only 0.16% after 72 h of oscillation incubation. It also showed significant biocompatibility, with a hemolysis rate and cell death rate of only 0.13% and 3.41%, respectively, compared to 14.72% and 70.11% for bare AC. According to ISO 10993 standards, this coated adsorbent material has low hemolysis and cytotoxicity (Zhang et al., 2018).

#### Heparin as a coating material for carbon-based adsorbents

Although Chang successfully developed a method using collodion and albumin to encapsulate charcoal for blood perfusion (Chang, 1975; Chang, 1976), and P. Lesche replicated this technique, Lesche’s attempt to integrate heparin into the encapsulation layer of charcoal particles to avoid localized heparinization was unsuccessful (Lesche et al., 1976). The reasons for the failure may be that the anticoagulant effect of heparin was weakened or inactivated after binding with collodion and albumin. Additionally, the binding stability of heparin with these materials could also be a problem, especially considering that heparin has a metabolic cycle of only 3–4 h in the human body, and this instability may exacerbate the reduction of anticoagulant effect. Although heparin anticoagulant coatings are widely used in medical materials (Biran and Pond, 2017), their application in blood adsorption materials remains limited due to unsolved key technical challenges. However, in recent years, there seems to have been some breakthroughs in this issue. In 2015, the Wei H team developed a heparin-modified chitosan/graphene oxide hybrid hydrogel (hep-CS/GH) using a freeze-drying, neutralization, and modification strategy for bilirubin adsorption. The prepared hybrid hydrogel exhibited a unique foam-like porous structure and excellent mechanical flexibility. After modifying the hydrogel with heparin, protein adsorption, platelet adhesion, and hemolysis were reduced, and the plasma coagulation time was extended from 4.1 min to 23.6 min, indicating that hep-CS/GH had good blood compatibility. Therefore, this study can lay a foundation for improving the performance of adsorbents in removing blood toxins (Wei et al., 2015). Ye Yang reported on the construction of Kevlar nanofiber/graphene oxide composite beads as safe, self-anticoagulant, and highly efficient hemoperfusion adsorbents. They first prepared Kevlar nanofiber-graphene oxide (K-GO) beads through liquid-liquid phase separation. Then, sodium p-styrenesulfonate (SS) was adsorbed onto the K-GO interface through π-π interactions and initiated to obtain composite gel (K-GO/PSS) beads with an interfacial cross-linked structure. These composite gel beads had excellent mechanical strength and self-anticoagulant ability due to their double network structure and heparin-like gel structure (Yang et al., 2020). In 2019, Qi Dang successfully fixed heparin as a molecular spacer onto microspheres, thereby significantly improving blood compatibility during blood perfusion (Dang et al., 2019). The research team deeply recognized the structural function of heparin as a macromolecular spacer to be crucial. Based on this, they used heparin as a spacer, covalently fixed it onto chloromethylated polystyrene microspheres (Ps), and further connected it with L-phenylalanine to construct a Ps-Hep-Phe structure, which performed well in the adsorption of endotoxins. When the initial concentration of the heparin solution was 5 mg/mL, the grafting density of heparin reached an optimum. The research results showed that the adsorbent with heparin as a spacer not only prolonged the coagulation time but also reduced protein adsorption and hemolysis rate, which fully proved that the heparin-modified adsorbent had excellent blood compatibility. In dynamic adsorption experiments, Ps-Hep-Phe had an adsorption capacity for endotoxins as high as 25.15 EU/g, which was significantly higher than that of Ps. In summary, this study strongly indicates that heparin has great potential as a modified material for adsorbents in blood perfusion.

In summary, carbon-based materials are still the most commonly used adsorption materials.

#### Silica-based materials

Silica-based materials, often in the form of nanoparticles, exhibit high specific surface area and large pore volume. These characteristics, coupled with their good mechanical stability, make them highly suitable for use in dialysate or plasma perfusion (Asano et al., 2008). However, early reports indicated that amino/methyl-modified silica exhibited a hemolysis rate exceeding 80% (Tang et al., 2011). In recent years, research on the biocompatibility of silica-based blood perfusion adsorbents has been insufficient. While modified silica particles have been used for bilirubin adsorption, their overall biocompatibility still requires further improvement.

### Characteristics and improvement of blood compatibility of organic adsorbents

#### Resin adsorbents

Resin adsorbents are widely used in blood purification due to their excellent chemical stability and adjustable pore structure (Rosenbaum et al., 1971; Raja, 1986). In the field of blood purification today, the use of adsorption materials derived from resins has become one of the most dominant methods (Asgharpour et al., 2020; Borazjani et al., 2023). These materials exhibit good plasticity and adjustability, allowing for the design of specific adsorption sites for particular toxins. However, a major challenge lies in nonspecific protein adsorption, which may reduce the material’s biocompatibility and therapeutic efficiency. Therefore, the research and development focus on resin materials primarily centers on modifying the resins to reduce nonspecific protein adsorption while enhancing their blood compatibility.

#### Human serum albumin (HSA) coating technology in resin adsorbents

In the early 1970 s, researchers conceived the idea of using albumin-coated resin materials to reduce blood damage. They employed a specialized extracorporeal circulation circuit to evaluate the blood compatibility of HSA-coated Amberlite XAD-7 resin. Within 2 hours after perfusion with uncoated resin, the average platelet loss (44 ± 5.6) % was greater than that after perfusion with HSA-coated resin (17 ± 2.2) % (*p* < 0.01). The average white blood cell loss was similar for both resins (55%). No increase in Swank filter pressure, which is used to detect the presence of cell aggregates in blood, was observed. Therefore, coating XAD-7 resin with HSA improved its blood compatibility in terms of platelet loss (Hughes et al., 1978). Subsequently, the team conducted *in vivo* experiments using HSA-coated XAD-7 resin for a single blood perfusion in four patients with acute liver failure. At the end of the 4-h blood perfusion, the average platelet count was (116 ± 16.3) % of the initial arterial value, and the average white blood cell count was (96 ± 6.5) % of the initial value. These results indicated that HSA-coated Amberlite XAD-7 resin was blood-compatible and capable of removing protein-bound substances and medium-sized molecules from the bodies of patients with acute liver failure (Hughes et al., 1979). However, due to the high cost of albumin as a coating material and the strict limitations on the production of human blood-derived albumin, it cannot be widely used clinically as a low-cost method. Therefore, there is a preference for finding adsorption materials with better blood compatibility, scalability, and cost-effectiveness.

While HSA coating showed promise, researchers continued to explore other coating technologies to improve blood compatibility and cost-effectiveness. One such technology is zwitterionic polymer (carboxybetaine) (PCB) hydrogel coating.

#### Zwitterionic polymer (carboxybetaine) (PCB) hydrogel coating in resin adsorbents

In Li et al. (2019) developed a blood adsorbent that is highly blood-compatible and effective, referred to as PCB-H103. This material was prepared by encapsulating polystyrene resin (H103) microparticles within a zwitterionic poly (carboxybetaine) (PCB) hydrogel matrix, which exhibits antifouling properties. To validate its performance, the research team not only tested the mechanical stability of PCB-H103 but also conducted thorough investigations into its adsorption efficiency in phosphate-buffered saline, bovine serum albumin solution, and 100% fetal bovine serum (FBS). Additionally, the team evaluated the blood compatibility of this novel adsorbent, PCB-H103. Benefiting from the antifouling capabilities of the PCB hydrogel, PCB-H103 exhibited excellent blood compatibility with a hemolysis rate of only approximately 0.64%. This result is significantly better than many existing blood adsorbent materials, indicating that PCB-H103 offers high safety and reliability in blood-contacting applications (Li et al., 2019).

#### Polymer-based materials (MIP)

Molecularly imprinted polymer-based materials (MIP) are special porous materials that contain recognition sites specific to certain toxin molecules, enabling highly selective adsorption of these molecules. Currently, MIP materials targeted at specific toxins have been successfully prepared and have shown a certain level of blood compatibility and adsorption efficacy. In Wu et al. (2017) combined electrospinning technology with molecular imprinting technology to prepare bilirubin-imprinted polydopamine on the surface of polyethersulfone (PES) sheets obtained through electrospinning, resulting in a bilirubin-imprinted polydopamine/PES composite material. This material induced minimal hemolysis, exhibited slight intrinsic anticoagulant activity, and had the ability to selectively adsorb bilirubin from cholesterol/bilirubin or testosterone/bilirubin biphasic solutions (Wu et al., 2017). In Osman et al. (2019) prepared uric acid-imprinted *P*(HEMA-MAC)-Fe^3+^ nanoparticles and subsequently polymerized these nanoparticles with acrylamide, methyl methacrylate, and methylene bisacrylamide at −18°C to obtain a cryogel. This cryogel did not induce significant coagulation and had a uric acid adsorption capacity of 148.6 mg·g^−1^ in serum (Osman et al., 2019), also demonstrating excellent blood compatibility. Research on MIP materials remains limited due to restricted clinical application scenarios, resulting in a paucity of related studies in this area.

#### Mixed matrix membrane (MMM) materials

Mixed matrix membrane (MMM) materials are membrane materials that contain embedded adsorbent particles, primarily achieving the adsorption process through these particles. In clinical practice, blood perfusion is often used in conjunction with hemodialysis; however, achieving a “smooth” transition between the two processes remains challenging. MMMs combine the “adsorption” process and the “diffusion” process, making it possible to integrate blood perfusion and hemodialysis into a single process. In Tijink et al. (2013) prepared hollow fiber MMMs using a dry-wet spinning technique based on immersion precipitation. These MMMs did not induce significant hemolysis and exhibited a certain level of cell compatibility and good mechanical stability. However, currently, research in this area is still in the exploratory stage.

#### Composite adsorbent materials

Composite adsorbent materials, which combine inorganic and organic materials, possess the advantages of both and have been widely used in blood perfusion technology in recent years. These composite materials not only exhibit efficient adsorption performance but also significantly enhance blood compatibility. Zhou W et al. designed and synthesized collagen (Col) and collagen-polyethyleneimine (Col-PEI) microspheres for bilirubin adsorption in patients (Zhou et al., 2023). Initially, pure collagen microspheres were synthesized through suspension polymerization using water-soluble carbodiimide (WSC) as a crosslinking agent. Subsequently, to improve the performance of Col microspheres, PEI was immobilized on their surface, forming collagen-PEI (Col-PEI) microspheres. The blood compatibility of the microspheres was assessed through routine blood tests, hemolysis tests, coagulation tests, and plasma recalcification time (PRT) tests. The results demonstrated that the hemolysis rate of the microspheres was less than 3%, which is lower than the standard set by ASTM (5%). Notably, the hemolysis rate of Col microspheres was only 0.61%, indicating excellent blood compatibility. Routine blood tests also showed no significant difference in the impact on blood cells compared to the control group. Compared to the control group, there was no significant decrease in APTT, PT, or TT levels for Col microspheres. However, the APTT of Col-PEI microspheres was significantly prolonged, indicating good anticoagulant capacity. Fibrinogen (FIB) is converted into insoluble fibrin under the action of enzymes, and fibrin is the main component of coagulation activation. The study found that the amount of FIB in Col-PEI microspheres was reduced compared to Col microspheres, which may be attributed to the enhanced anticoagulant capacity of Col-PEI microspheres due to PEI grafting. The PRT test assessed the anticoagulant capacity of the microspheres, and the results showed that the PRT of Col microspheres and Col-PEI microspheres was 2.30 min and 3.1 min, respectively, which was consistent with that of Col microspheres. Based on the results of biocompatibility and blood compatibility, the researchers believed that microspheres designed based on collagen are suitable for blood perfusion applications.

In Yang et al. (2021) prepared novel polymyxin B (PMB) engineered polystyrene-divinylbenzene microspheres. PMB is a cyclic and highly cationic decapeptide extracted from *Bacillus* polymyxa, and the endotoxin-PMB complex is very stable with an association constant (Ka) ranging from 1.8 × 10^−6^ to 2.3 × 10^−6^ M^−1^. The research team successfully grafted 11-mercaptoundecanoic acid (MA) onto the microspheres using thiol-ene click chemistry as a linker and then immobilized PMB on the surface of the microspheres through EDC/NHS coupling reaction.

To verify the biosafety of these engineered microspheres, the research team successfully grafted MA onto the microspheres using thiol-ene click chemistry as a linker. Subsequently, they immobilized PMB on the surface of the microspheres through an EDC/NHS coupling reaction. To verify the biosafety of these engineered microspheres, the research team conducted a series of biocompatibility and blood compatibility tests. The Cell Counting Kit-8 assay (CCK-8) revealed that the cytotoxicity of P-PMB microsphere extracts towards L929 and HUVEC cells was negligible compared to the culture medium, similar to PS-DVB and P-MA microspheres, demonstrating their excellent biocompatibility.

In blood compatibility tests, the team initially performed a hemolysis test. The results indicated no visible hemolysis in the P-PMB microsphere extracts. Quantitative analysis further confirmed a hemolysis rate of only 2.20% for P-PMB microspheres, indicating good blood compatibility according to the ISO 10993-5:1992 standard (hemolysis rate less than 5%). Anticoagulation tests further substantiated the blood compatibility of the microspheres. The *in vitro* coagulation time tests showed that the APTT of the P-PMB microsphere group was slightly lower than that of the PS-DVB group, though the difference was not statistically significant. Similarly, there were no significant differences in PT and TT between the original PS-DVB and engineered P-PMB microspheres, suggesting that the engineered microspheres did not adversely affect endogenous coagulation. Furthermore, they also found that both the original microspheres (PS-DVB) and the engineered microspheres (P-MA and P-PMB) had no effect on the adsorption of fibrinogen (FIB). These results collectively demonstrated the biosafety of PMB engineered microspheres during application and showed their great potential as blood-contacting materials in blood perfusion.

Additionally, materials such as MXenes and metal-organic frameworks (MOFs), as novel composite adsorbents, have received widespread attention in blood perfusion technology in recent years due to their highly tunable pore structures and excellent adsorption performance (Naguib et al., 2011). MXenes stand out as typical representatives of novel adsorbents with their unique two-dimensional nanostructure and good biocompatibility (Meng et al., 2018). They exhibit excellent biological and blood compatibility and can effectively adsorb and remove urea from aqueous solutions and dialysis fluids. MOFs, on the other hand, demonstrate remarkable adsorption performance due to their high specific surface area, tunable pore size, and functional diversity, particularly in adsorbing toxins such as p-cresyl sulfate, indoxyl sulfate, and hippuric acid (Zhao et al., 2020; Yao et al., 2023). Hemolysis tests, coagulation time tests, and cytotoxicity tests have confirmed that bilirubin adsorbents based on UiO-66 and UiO-66-NH2 exhibit good blood compatibility and biocompatibility (Liu et al., 2023). Since MXenes and MOFs are emerging materials in recent years, research on their blood compatibility is still limited due to immature technology.

#### Polysaccharide-based adsorbent materials

Polysaccharide-based adsorbents primarily include agarose, chitosan, and cellulose (Wu et al., 2019; Li et al., 2023). These materials exhibit excellent biocompatibility and adsorption properties, effectively removing harmful substances from the blood. Due to their high modifiability, chemical modifications of these polysaccharide-based adsorbents can further enhance their adsorption selectivity for specific substances, thereby improving the effectiveness of blood perfusion. Wang Y et al. developed an advanced technology for carboxymethyl chitosan-based heparin-mimetic cross-linked bead adsorbents (Wang et al., 2018). The team initially used safe and efficient carboxymethyl chitosan as the raw material and then utilized hydrogen bonding interactions for *in-situ* crosslinking with 2-acrylamido-2-methyl-1-propanesulfonic acid (AMPS) to prepare a polymer molecular adsorbent using heparin as the raw material for low-density lipoprotein-cholesterol (LDL-C) removal during blood purification. They employed various methods such as Fourier transform infrared spectroscopy, two-dimensional correlation infrared spectroscopy, thermogravimetric analysis, and energy-dispersive X-ray spectroscopy (EDS) to confirm the feasibility of this technology. Due to their good hydrophilicity, these beads exhibit excellent blood compatibility when in contact with blood. The research results demonstrate that the hemolysis rate of all beads is less than 5%, and the platelet aggregation time for ccm/PAMPS beads exceeds 600 s, effectively inhibiting contact activation and supplementary activation. Furthermore, these microbeads do not exhibit significant cytotoxicity towards endothelial cells, making them a safe adsorbent for blood purification. Song et al. (2018) also designed “Design of Carrageenan-Based Heparin-Mimetic Gel Beads as Self-Anticoagulant Hemoperfusion Adsorbents”. Based on natural polysaccharides, this process uses carrageenan phase inversion technology combined with post-crosslinking treatment with polyacrylic acid (PAA) to construct a stable cross-linked network between carrageenan and polyacrylic acid. Experimental data show that this material has a high adsorption capacity for exogenous toxins such as heavy metal ions, reaching up to 560.34 mg/g, and also exhibits significant adsorption effects on endogenous toxins such as creatinine, bilirubin, and low-density lipoprotein, achieving adsorption capacities of 14.83 mg/g, 228.16 mg/g, and 18.15 mg/g, respectively. Additionally, its low protein adsorption capacity, low hemolysis rate, and low cytotoxicity further demonstrate its advantages in biocompatibility. It is particularly worth mentioning that this novel adsorbent exhibits exceptional performance in coagulation. Experimental results indicate that the activated partial thromboplastin time, prothrombin time, and thrombin time of the gel beads are significantly higher than those of the control group, with respective extensions of 13 times, 18 times, and four times. This finding suggests that the material has excellent self-anticoagulant properties, which can significantly reduce the risk of coagulation during blood perfusion, thereby enhancing the safety and efficiency of treatment. This characteristic makes the material highly promising for applications in the field of blood purification. To clearly understand the coating methods used for hemoperfusion adsorbent materials and their advantages and disadvantages, the author has created a simple table to more clearly express the differences and advantages of different coating technologies (see Table 1).

**TABLE 1:** A summary of adsorbent materials and coating technologies for enhanced blood compatibility in hemoperfusion applications.

Substrate type	Coating technology	Principle	Major advantages	Limitations	Representative materials	Application of universal strategies
Carbon-based	Hydrogel or Polymer Coating	Coating a thin film on activated carbon particles	Improves blood compatibility, reduces carbon particle shedding	Increases production cost, requires uniform and stable coating	Albumin collodion, PAA hydrogel, PVA	Lecithin coating to enhance biocompatibility and functionality
Carbon-based	Microcapsule Technology	Coating a layer with strength and permeability on activated carbon	Improves shedding and poor blood compatibility of carbon particles	Complex process, requires suitable capsule material for biocompatibility and permeability	Albumin collodion, PMC, PAA hydrogel	Suitable for porous materials, controlled release, and targeted delivery
Carbon-based	Mixing Activated Carbon with Polymers	Mixing activated carbon powder with other hydrophilic polymers	Combines advantages, improves overall performance	Requires uniform distribution, precise control of mixing ratio	PVA, PAA, PEG	Combines advantages of different materials, enhances adsorption performance
Carbon-based	Surface Modification or Composite Adsorbents	Introducing functional groups or bioactive ligands on activated carbon	Provides more functions and specific adsorption capacity	Requires precise control of component ratios and reaction conditions	PEG, silica gel, functional groups	Combines characteristics of different materials, enhances functionality and specific adsorption
Silicon-based	Nano-particle Modification	Surface modification of nano-scale SiO2 particles	High specific surface area and pore volume, good mechanical stability	Needs further improvement in biocompatibility	SiO_2_ nanoparticles	Tannic acid derivatives with amino groups to enhance biocompatibility
Resin-based	Human Serum Albumin Coating	Coating human serum albumin on resin surface	Improves blood compatibility, reduces platelet loss	High cost, limited albumin source	Polystyrene resin (H103)	Surface modification with high molecular weight surfactants for uniform particle deposition
Resin-based	Zwitterionic Polymer Hydrogel Coating	Encapsulating resin particles in anti-biofouling zwitterionic hydrogel	Excellent biocompatibility and anti-fouling ability	Complex coating preparation process	H103 resin microparticles encapsulated in PCB hydrogel	Dopamine-mediated zwitterionic polyelectrolyte coating to reduce fibrosis and residual hearing loss
Polymer-based	Molecular Imprinting Technology	Electrospinning combined with molecular imprinting to prepare specific recognition sites	High selectivity adsorption, good blood compatibility	Limited clinical applications, relatively few studies	Bilirubin imprinted polydopamine/polyethersulfone composite materials	Designing high selectivity adsorbents for specific toxins
Mixed Matrix Membranes	Embedding Adsorbent Particles in Membranes	Achieving adsorption by embedding adsorbent particles	Combines “adsorption” and “diffusion”, promising potential	Research is still in the exploration stage	Not specified	Exploring integrated “adsorption” and “diffusion” processes
Novel Composite Adsorbents	Inorganic-Organic Composites	Combining inorganic and organic materials for combined advantages	High adsorption performance and excellent blood compatibility	Technology relatively immature, needs further research	Collagen (Col) and Col-PEI microspheres	Combining advantages of different materials, enhances overall performance
Polysaccharide Adsorbents	Chemically Modified Polysaccharide Materials	Improving adsorption selectivity and biocompatibility through chemical modification	Excellent biocompatibility and adsorption performance	Complex modification process, requires optimization of modification conditions	Heparin-mimetic crosslinked beads based on carboxymethyl chitosan, heparin-mimetic gel beads based on carrageenan	Lecithin coating to enhance stability and functionality

PAA, polyacrylic acid; PVA, polyvinyl alcohol; PMC, poly methyl cellulose; PEG, polyethylene glycol.

## Conclusion

Recent advancements in blood purification adsorbents have significantly improved their blood compatibility. A variety of materials—including carbon-based, silica-based, resin, molecularly imprinted polymers, mixed matrix membranes, novel composites, and polysaccharide adsorbents—have been extensively researched and optimized through advanced coating techniques. These innovations have enhanced the safety and efficacy of blood purification in clinical applications, reducing adverse reactions.

However, challenges remain. Complex fabrication processes and high production costs hinder the widespread clinical adoption of several innovative materials. Additionally, while laboratory studies show promising results, the long-term stability and safety of these materials in real-world clinical settings require further validation.

Looking ahead, the field of blood purification adsorbents presents significant opportunities for growth and innovation. Key research directions include.

1) Cost-Effectiveness and Scalability: Developing affordable and scalable manufacturing processes is essential for broader clinical use, especially in resource-limited settings.
2) Understanding Blood-Material Interactions: A deeper understanding of the mechanisms governing these interactions will aid in designing materials with superior blood compatibility.
3) Interdisciplinary Collaboration: Encouraging collaboration among materials scientists, biomedical engineers, and clinicians can accelerate the translation of laboratory findings into clinical applications.
4) Emerging Technologies: Investigating the potential of emerging technologies, such as metal-organic frameworks (MOFs) and biomimetic coatings, offers promising avenues for enhancing performance and compatibility.

In conclusion, achieving optimal blood compatibility in purification adsorbents requires ongoing interdisciplinary collaboration. By addressing current challenges and leveraging emerging technologies, we anticipate that future advancements will lead to safer and more effective blood purification therapies for patients worldwide.

## References

[B1] Andrade J. D. Kunitomo K. Van Wagenen R. Kastigir B. Gough D. Kolff W. J. (1971). Coated adsorbents for direct blood perfusion: HEMA-activated carbon. Trans. - Am. Soc. Artif. Intern. Organs 17, 222–228. 5158096

[B2] Asano T. Tsuru K. Hayakawa S. Osaka A. (2008). Bilirubin adsorption property of sol-gel-derived titania particles for blood purification therapy. Acta Biomater. 4, 1067–1072. 10.1016/j.actbio.2008.02.024 18381253

[B3] Asgharpour M. Mehdinezhad H. Bayani M. Zavareh M. Hamidi S. H. Akbari R. (2020). Effectiveness of extracorporeal blood purification (hemoadsorption) in patients with severe coronavirus disease 2019 (COVID-19). BMC Nephrol. 21, 356. 10.1186/s12882-020-02020-3 32819292 PMC7439633

[B4] Bale S. Khurana A. Reddy A. S. Singh M. Godugu C. (2016). Overview on therapeutic applications of microparticulate drug delivery systems. Crit. Rev. Ther. Drug Carr. Syst. 33, 309–361. 10.1615/CritRevTherDrugCarrierSyst.2016015798 27910739

[B5] Bhattacharjee A. Savargaonkar A. V. Tahir M. Sionkowska A. Popat K. C. (2024). Surface modification strategies for improved hemocompatibility of polymeric materials: a comprehensive review. RSC Adv. 14, 7440–7458. 10.1039/d3ra08738g 38433935 PMC10906639

[B6] Biran R. Pond D. (2017). Heparin coatings for improving blood compatibility of medical devices. Adv. Drug Deliv. Rev. 112, 12–23. 10.1016/j.addr.2016.12.002 28042080

[B7] Borazjani R. Mahmudi-Azer S. Taghrir M. H. Homaeifar R. Dabiri G. Paydar S. (2023). Adjunctive hemoperfusion with Resin Hemoadsorption (HA) 330 cartridges improves outcomes in patients sustaining multiple Blunt Trauma: a prospective, quasi-experimental study. BMC Surg. 23, 148. 10.1186/s12893-023-02056-w 37270595 PMC10239212

[B8] Bowry S. K. Kircelli F. Himmele R. Nigwekar S. U. (2021). Blood-incompatibility in haemodialysis: alleviating inflammation and effects of coagulation. Clin. Kidney J. 14, i59–i71. 10.1093/ckj/sfab185 34987786 PMC8711760

[B9] Brash J. L. Horbett T. A. Latour R. A. Tengvall P. (2019). The blood compatibility challenge. Part 2: protein adsorption phenomena governing blood reactivity. Acta Biomater. 94, 11–24. 10.1016/j.actbio.2019.06.022 31226477 PMC6642842

[B10] Buhrman J. S. Cook L. C. Rayahin J. E. Federle M. J. Gemeinhart R. A. (2013). Proteolytically activated anti-bacterial hydrogel microspheres. J. Control Release 171, 288–295. 10.1016/j.jconrel.2013.06.023 23816641 PMC3795988

[B11] Cai N. Li Q. Zhang J. Xu T. Zhao W. Yang J. (2017). Antifouling zwitterionic hydrogel coating improves hemocompatibility of activated carbon hemoadsorbent. J. Colloid Interface Sci. 503, 168–177. 10.1016/j.jcis.2017.04.024 28521219

[B12] Camire R. M. (2021). Blood coagulation factor X: molecular biology, inherited disease, and engineered therapeutics. J. Thromb. Thrombolysis 52, 383–390. 10.1007/s11239-021-02456-w 33886037 PMC8531165

[B13] Chandy T. Sharma C. P. (1993). Preparation and performance of chitosan encapsulated activated charcoal (ACCB) adsorbents for small molecules. J. Microencapsul. 10, 475–486. 10.3109/02652049309015324 8263676

[B14] Chang T. M. (1975). Microencapsulated adsorbent hemoperfusion for uremia, intoxication and hepatic failure. Kidney Int., 387–392. 1099317

[B15] Chang T. M. (1976). Microcapsule artificial kidney: including updated preparative procedures and properties. Kidney Int., S218–S224. 1070536

[B16] Chang T. M. (1984). Coated charcoal haemoperfusion. Life support Syst. J. Eur. Soc. Artif. Organs 2, 99–106. 6384672

[B17] Cheah W. K. Ishikawa K. Othman R. Yeoh F. Y. (2017). Nanoporous biomaterials for uremic toxin adsorption in artificial kidney systems: a review. J. Biomed. Mat. Res. Part B Appl. Biomater. 105, 1232–1240. 10.1002/jbm.b.33475 26913694

[B18] Chen D. Wang X. Liang J. Zhang Z. Chen W. (2021). A novel electrospinning polyacrylonitrile separator with dip-coating of zeolite and phenoxy resin for Li-ion batteries. Membr. (Basel) 11, 267. 10.3390/membranes11040267 PMC8068060 33917680

[B19] Damianaki A. Stambolliu E. Alexakou Z. Petras D. (2023). Expanding the potential therapeutic options of hemoperfusion in the era of improved sorbent biocompatibility. Kidney Res. Clin. Pract. 42, 298–311. 10.23876/j.krcp.22.223 37098671 PMC10265212

[B20] Dang Q. Li C. G. Jin X. X. Zhao Y. J. Wang X. (2019). Heparin as a molecular spacer immobilized on microspheres to improve blood compatibility in hemoperfusion. Carbohydr. Polym. 205, 89–97. 10.1016/j.carbpol.2018.08.067 30446153

[B21] Davenport A. (2017). New dialysis technology and biocompatible materials. Contrib. Nephrol. 189, 130–136. 10.1159/000450739 27951560

[B22] Denti E. Luboz M. P. Tessore V. (1975). Adsorption characteristics of cellulose acetate coated charcoals. J. Biomed. Mater. Res. 9, 143–150. 10.1002/jbm.820090204 1176475

[B23] Dong X. Sun Q. Geng J. Liu X. Wei Q. (2024). Fiber flexibility reconciles matrix recruitment and the fiber modulus to promote cell mechanosensing. Nano Lett. 24, 4029–4037. 10.1021/acs.nanolett.4c00923 38526438

[B24] Erturk E. Haberal M. Piskin E. (1987). Towards the commercialization of hemoperfusion column. Part II. Coating of activated carbon. Biomaterials, Artif. cells, Artif. organs 15, 633–654. 10.3109/10731198709117560 3440136

[B25] Gan N. Sun Q. Zhao L. Zhang S. Suo Z. Wang X. (2021). Hierarchical core-shell nanoplatforms constructed from Fe(3)O(4)@C and metal-organic frameworks with excellent bilirubin removal performance. J. Mater Chem. B 9, 5628–5635. 10.1039/d1tb00586c 34109969

[B26] Gao C. Zhang Q. Yang Y. Li Y. Lin W. (2022). Recent trends in therapeutic application of engineered blood purification materials for kidney disease. Biomater. Res. 26, 5. 10.1186/s40824-022-00250-0 35120554 PMC8815201

[B27] Gupta T. K. Budarapu P. R. Chappidi S. R. Y B S. S. Paggi M. Bordas S. P. (2019). Advances in carbon based nanomaterials for bio-medical applications. Curr. Med. Chem. 26, 6851–6877. 10.2174/0929867326666181126113605 30474523

[B28] He S. Cao H. Thålin C. Svensson J. Blombäck M. Wallén H. (2021). The clotting trigger is an important determinant for the coagulation pathway *in vivo* or *in vitro*-Inference from data review. Semin. Thromb. Hemost. 47, 063–073. 10.1055/s-0040-1718888 33348413

[B29] Hughes R. Ton H. Y. Langley P. Davies M. Hanid M. A. Mellon P. (1979). Albumin-coated Amberlite XAD-7 resin for hemoperfusion in acute liver failure. Part II: *in vivo* evaluation. Artif. Organs 3, 23–26. 10.1111/j.1525-1594.1979.tb03800.x 435120

[B30] Hughes R. D. Ton H. Y. Langley P. G. Silk D. B. Williams R. (1978). The use of an *in vitro* haemoperfusion circuit to evaluate the blood compatibility of albumin-coated Amberlite XAD-7 resin. Int. J. Artif. Organs 1, 129–134. 689755

[B31] Jo Y. K. Seo J. H. Choi B. H. Kim B. J. Shin H. H. Hwang B. H. (2014). Surface-independent antibacterial coating using silver nanoparticle-generating engineered mussel glue. ACS Appl. Mater. and Interfaces 6, 20242–20253. 10.1021/am505784k 25311392

[B32] Kuchinka J. Willems C. Telyshev D. V. Groth T. (2021). Control of blood coagulation by hemocompatible material surfaces-A review. Bioeng. (Basel) 8, 215. 10.3390/bioengineering8120215 PMC8698751 34940368

[B33] Lesche P. Blume U. Scheibe G. Sussmann P. Schmidt F. W. Bartels M. (1976). Hemoperfusion by means of encapsuled charcoal for the treatment of exogenous and endogenous intoxications. Klin. Wochenschr. 54, 509–516. 10.1007/BF01468971 933456

[B34] Li M. Chen M. Yang F. Qin R. Yang Q. Ren H. (2023). Protein/polysaccharide composite toward multi-in-one toxin removal in blood with self-anticoagulation and biocompatibility. Adv. Healthc. Mater 12, e2300999. 10.1002/adhm.202300999 37334878

[B35] Li Q. Yang J. Cai N. Zhang J. Xu T. Zhao W. (2019). Hemocompatible hemoadsorbent for effective removal of protein-bound toxin in serum. J. Colloid Interface Sci. 555, 145–156. 10.1016/j.jcis.2019.07.045 31377640

[B36] Lie T. S. Kim W. I. Rommelsheim K. Holst A. (1976). Treatment of comatose patients by extracorporeal hemoperfusion with activated charcoal. MMW, Munchener Med. Wochenschr. 118, 945–948. 820984

[B37] Liu Y. Yuan Z. Chen Y. (2023). Metal-organic framework (UiO-66 and UiO-66-NH(2))-based adsorbents for bilirubin removal used in hemoperfusion. RSC Adv. 13, 35078–35087. 10.1039/d3ra07212f 38046623 PMC10691446

[B38] Ma G. (2014). Microencapsulation of protein drugs for drug delivery: strategy, preparation, and applications. J. Control Release 193, 324–340. 10.1016/j.jconrel.2014.09.003 25218676

[B39] Maitz M. F. Martins M. Grabow N. Matschegewski C. Huang N. Chaikof E. L. (2019). The blood compatibility challenge. Part 4: surface modification for hemocompatible materials: passive and active approaches to guide blood-material interactions. Acta Biomater. 94, 33–43. 10.1016/j.actbio.2019.06.019 31226481

[B40] Meng F. Seredych M. Chen C. Gura V. Mikhalovsky S. Sandeman S. (2018). MXene sorbents for removal of urea from dialysate: a step toward the wearable artificial kidney. ACS Nano 12, 10518–10528. 10.1021/acsnano.8b06494 30257087

[B41] Naguib M. Kurtoglu M. Presser V. Lu J. Niu J. Heon M. (2011). Two-dimensional nanocrystals produced by exfoliation of Ti3 AlC2. Adv. Mater. Deerf. Beach, Fla 23, 4248–4253. 10.1002/adma.201102306 21861270

[B42] Nakane M. (2004). Basics of hemoadsorption. Nihon rinsho. Jpn. J. Clin. Med. 62 (Suppl. 5), 22–28. 15197882

[B43] Osman B. Sagdilek E. Gümrükçü M. Göçenoğlu Sarıkaya A. (2019). Molecularly imprinted composite cryogel for extracorporeal removal of uric acid. Colloids Surf. B Biointerfaces 183, 110456. 10.1016/j.colsurfb.2019.110456 31472391

[B44] Pagel M. Beck-Sickinger A. G. (2017). Multifunctional biomaterial coatings: synthetic challenges and biological activity. Biol. Chem. 398, 3–22. 10.1515/hsz-2016-0204 27636830

[B45] Patel H. (2021). Blood biocompatibility enhancement of biomaterials by heparin immobilization: a review. Blood Coagul. Fibrinolysis 32, 237–247. 10.1097/MBC.0000000000001011 33443929

[B46] Pişkin E. Cakmakli C. Ozdural A. R. (1981). The best coating material for hemoperfusion: comparation of cellulose nitrate with cellulose acetate and derivatives. Int. J. Artif. Organs 4, 86–88. 10.1177/039139888100400213 7275339

[B47] Raja R. M. (1986). Resin hemoperfusion for drug intoxication--an update. Int. J. Artif. Organs 9, 319–322. 10.1177/039139888600900511 2430899

[B48] Redolfi Riva E. D'Alessio A. Micera S. (2022). Polysaccharide layer-by-layer coating for polyimide-based neural interfaces. Micromachines (Basel) 13, 692. 10.3390/mi13050692 35630159 PMC9146946

[B49] Ren X. Feng Y. Guo J. Wang H. Li Q. Yang J. (2015). Surface modification and endothelialization of biomaterials as potential scaffolds for vascular tissue engineering applications. Chem. Soc. Rev. 44, 5680–5742. 10.1039/c4cs00483c 26023741

[B50] Ricci Z. Romagnoli S. Reis T. Bellomo R. Ronco C. (2022). Hemoperfusion in the intensive care unit. Intensive Care Med. 48, 1397–1408. 10.1007/s00134-022-06810-1 35984473 PMC9389493

[B51] Riesenfeld J. Olsson P. Sanchez J. Mollnes T. E. (1995). Surface modification with functionally active heparin. Med. device Technol. 6, 24–31. 10155375

[B52] Rokosz K. Hryniewicz T. Dudek Ł. (2020). Phosphate porous coatings enriched with selected elements via peo treatment on titanium and its alloys: a review. Mater. Basel, Switz. 13, 2468. 10.3390/ma13112468 PMC7321118 32481746

[B53] Ronco C. Bellomo R. (2022). Hemoperfusion: technical aspects and state of the art. Crit. Care London, Engl. 26, 135. 10.1186/s13054-022-04009-w PMC9097563 35549999

[B54] Rosenbaum J. L. Kramer M. S. Raja R. Boreyko C. (1971). Resin hemoperfusion: a new treatment for acute drug intoxication. N. Engl. J. Med. 284, 874–877. 10.1056/NEJM197104222841603 5549829

[B55] Sharma C. P. (2001). Blood-compatible materials: a perspective. J. Biomater. Appl. 15, 359–381. 10.1106/YY8L-M4DD-AGW1-QAGU 11336389

[B56] Song X. Ji H. Li Y. Xiong Y. Qiu L. Zhong R. (2021). Transient blood thinning during extracorporeal blood purification via the inactivation of coagulation factors by hydrogel microspheres. Nat. Biomed. Eng. 5, 1143–1156. 10.1038/s41551-020-00673-x 33495638

[B57] Song X. Wang K. Tang C. Q. Yang W. W. Zhao W. F. Zhao C. S. (2018). Design of carrageenan-based heparin-mimetic gel beads as self-anticoagulant hemoperfusion adsorbents. Biomacromolecules 19, 1966–1978. 10.1021/acs.biomac.7b01724 29425448

[B58] Sun W. Liu W. Wu Z. Chen H. (2020). Chemical surface modification of polymeric biomaterials for biomedical applications. Macromol. Rapid Commun. 41, e1900430. 10.1002/marc.201900430 32134540

[B59] Tang T. Li X. Xu Y. Wu D. Sun Y. Xu J. (2011). Bilirubin adsorption on amine/methyl bifunctionalized SBA-15 with platelet morphology. Colloids Surf. B Biointerfaces 84, 571–578. 10.1016/j.colsurfb.2011.02.019 21382700

[B60] Tijink M. S. Wester M. Glorieux G. Gerritsen K. G. Sun J. Swart P. C. (2013). Mixed matrix hollow fiber membranes for removal of protein-bound toxins from human plasma. Biomaterials 34, 7819–7828. 10.1016/j.biomaterials.2013.07.008 23876759

[B61] Ursino H. Zhang B. Ludtka C. Webb A. Allen J. B. (2022). Hemocompatibility of all-trans retinoic acid-loaded citrate polymer coatings for vascular stents. Regen. Eng. Transl. Med. 8, 579–592. 10.1007/s40883-022-00257-y 36714809 PMC9881644

[B62] Wang X. Yan X. Wang B. C. Xue W. Zhou C. R. Zhang Y. (2021). TMPyP-bound guanosine-borate supramolecular hydrogel as smart hemoperfusion device with real-time visualized/electrochemical bi-modal monitoring for selective blood lead elimination. Biosens. Bioelectron. 184, 113230. 10.1016/j.bios.2021.113230 33872980

[B63] Wang Y. Huang X. He C. Li Y. Zhao W. Zhao C. (2018). Design of carboxymethyl chitosan-based heparin-mimicking cross-linked beads for safe and efficient blood purification. Int. J. Biol. Macromol. 117, 392–400. 10.1016/j.ijbiomac.2018.05.091 29777805

[B64] Weber M. Steinle H. Golombek S. Hann L. Schlensak C. Wendel H. P. (2018). Blood-contacting biomaterials: *in vitro* evaluation of the hemocompatibility. Front. Bioeng. Biotechnol. 6, 99. 10.3389/fbioe.2018.00099 30062094 PMC6054932

[B65] Wei H. Han L. Tang Y. Ren J. Zhao Z. Jia L. (2015). Highly flexible heparin-modified chitosan/graphene oxide hybrid hydrogel as a super bilirubin adsorbent with excellent hemocompatibility. J. Mater Chem. B 3, 1646–1654. 10.1039/c4tb01673d 32262437

[B66] Wei Q. Haag R. (2015). Universal polymer coatings and their representative biomedical applications. Mater. HorizonsMater. Horiz. 2, 567–577. 10.1039/C5MH00089K

[B67] Wen K. C. Li Z. A. Liu J. H. Zhang C. Zhang F. Li F. Q. (2024). Recent developments in ureteral stent: substrate material, coating polymer and technology, therapeutic function. Colloids Surf. B Biointerfaces 238, 113916. 10.1016/j.colsurfb.2024.113916 38636438

[B68] Wu K. Liu X. Li Z. Jiao Y. Zhou C. (2020). Fabrication of chitosan/graphene oxide composite aerogel microspheres with high bilirubin removal performance. Mater Sci. Eng. C Mater Biol. Appl. 106, 110162. 10.1016/j.msec.2019.110162 31753385

[B69] Wu K. Yang W. Jiao Y. Zhou C. (2017). A surface molecularly imprinted electrospun polyethersulfone (PES) fiber mat for selective removal of bilirubin. J. Mater Chem. B 5, 5763–5773. 10.1039/c7tb00643h 32264210

[B70] Wu S. Yue P. Ma Y. Zou Y. Liang W. Ye Q. (2023). Hemoperfusion adsorbents for removal of common toxins in liver and kidney failure: recent progress, challenges, and prospects. Adv. Mater. Deerf. Beach, Fla, e2305152. 10.1002/adma.202305152 37566803

[B71] Wu Z. Deng W. Zhou W. Luo J. (2019). Novel magnetic polysaccharide/graphene oxide @Fe(3)O(4) gel beads for adsorbing heavy metal ions. Carbohydr. Polym. 216, 119–128. 10.1016/j.carbpol.2019.04.020 31047048

[B72] Xing X. Han Y. Cheng H. (2023). Biomedical applications of chitosan/silk fibroin composites: a review. Int. J. Biol. Macromol. 240, 124407. 10.1016/j.ijbiomac.2023.124407 37060984

[B73] Xiong J. Ye W. Mu L. Lu X. Zhu J. (2024). Separation of mono-/divalent ions via controlled dynamic adsorption/desorption at polythiophene coated carbon surface with flow-electrode capacitive deionization. Small 20, e2400288. 10.1002/smll.202400288 38593337

[B74] Yang K. Peng Y. Wang L. Ren L. (2021). Polymyxin B engineered polystyrene-divinylbenzene microspheres for the adsorption of bilirubin and endotoxin. RSC Adv. 11, 39978–39984. 10.1039/d1ra06684f 35494100 PMC9044794

[B75] Yang Y. Yin S. He C. Wu X. Yin J. Zhang J. (2020). Construction of Kevlar nanofiber/graphene oxide composite beads as safe, self-anticoagulant, and highly efficient hemoperfusion adsorbents. J. Mater Chem. B 8, 1960–1970. 10.1039/c9tb02789k 32067017

[B76] Yao M. Zhang G. Shao D. Ding S. Li L. Li H. (2023). Preparation of chitin/MXene/poly(L-arginine) composite aerogel spheres for specific adsorption of bilirubin. Int. J. Biol. Macromol. 243, 125140. 10.1016/j.ijbiomac.2023.125140 37270125

[B77] Zhang L. Hu G. Du Y. Gao L. Qi H. (2018). A facile modification to improve the biocompatibility and adsorbability of activated carbon with zwitterionic hydrogel. J. Mater Sci. Mater Med. 29, 113. 10.1007/s10856-018-6127-4 30019317

[B78] Zhang M. Liu X. Li X. Zhou W. Yu H. Wang S. (2023). A novel recyclable hemoperfusion adsorbent based on TiO_2_nanotube arrays for the selective removal of β_2_-microglobulin. J. Mater Chem. B 11, 7739–7749. 10.1039/d3tb01037f 37470708

[B79] Zhao Q. Seredych M. Precetti E. Shuck C. E. Harhay M. Pang R. (2020). Adsorption of uremic toxins using Ti_3_C_2_T*<sub><i>x*</sub></i> MXene for dialysate regeneration. ACS Nano 14, 11787–11798. 10.1021/acsnano.0c04546 32830949 PMC7530082

[B80] Zhao Y. Yuan P. Q. Xu X. R. Yang J. (2023). Removal of phosphate by adsorption with 2-phenylimidazole-modified porous ZIF-8: powder and chitosan spheres. ACS Omega 8, 28436–28447. 10.1021/acsomega.3c02671 37576661 PMC10413465

[B81] Zhou W. Hu W. Zhan Q. Zhang M. Liu X. Hussain W. (2023). Novel hemoperfusion adsorbents based on collagen for efficient bilirubin removal - a thought from yellow skin of patients with hyperbilirubinemia. Int. J. Biol. Macromol. 253, 127321. 10.1016/j.ijbiomac.2023.127321 37820900

